# Photochemical and Thermal Stability of Bionanocellulose/Poly(Vinyl Alcohol) Blends

**DOI:** 10.3390/polym14204364

**Published:** 2022-10-16

**Authors:** Aldona Długa, Dagmara Bajer, Halina Kaczmarek

**Affiliations:** 1 Bowil Biotech Sp. z.o.o., 7 Skandynawska St., 84-120 Władysławowo, Poland; 2 Faculty of Chemistry, Nicolaus Copernicus University in Toruń, 7 Gagarina St., 87-100 Toruń, Poland

**Keywords:** bionanocellulose, polymer blends, poly(vinyl alcohol), thermogravimetry, UV-irradiation

## Abstract

This research focuses on novel ecological materials for biomedical and cosmetic applications. The cellulose of bacterial origin is well suited for such purposes, but its functional properties must be modified. In this work, the blends of bionanocellulose and poly(vinyl alcohol), BNC/PVA, were prepared based on in situ and ex situ methodology combined with impregnation and sterilization, using different concentrations of PVA. The main purpose of this work was to check the influence of UV radiation and high temperature, which can be sterilizing factors, on the properties of these mixtures. It was found that the crystallinity degree increases in UV-irradiated samples due to the photodegradation of the amorphous phase. This changes the mechanical properties: the breaking stress and Young’s modulus decreased, while the strain at break increased in most UV-irradiated samples. The surface morphology, which we observed by using AFM, did not change significantly after exposure, but the roughness and surface free energy changed irregularly in samples obtained by different methods. However, the effects induced by UV-irradiation were not so crucial as to deteriorate the materials’ properties designed for medical applications. Thermogravimetric analysis exhibited good thermal stability for all samples up to at least 200 °C, which allows for the prediction of these systems also in industrial sectors.

## 1. Introduction

A significant challenge today is the development of the polymer industry toward replacing the ubiquitous products obtained from crude oil with alternative raw materials of natural origin. The aim is to increase the use of degradable materials in various sectors of the economy, as doing so may contribute to the recovery of the environmental balance currently disturbed by the increasing pollution and accumulation of plastic waste [1].

Biodegradable polymers such as cellulose, starch, and chitosan are excellent alternatives to polyolefins, polystyrene, poly(vinyl chloride), and other commonly used synthetic macromolecular compounds [2]. In addition to bio-sourced polymers, and synthetic biodegradable polymers such as polylactide, polyhydroxyalkanoates, polycaprolactone, polyglycolide, poly(butylene succinate), poly(ethylene adipate, poly(p-dioxanone), or poly(vinyl alcohol) are also increasingly utilized today [3].

These environmentally friendly materials are mainly valuable for biomedical (as scaffolds, implants, and biosensors), pharmaceutical (drug- or gene-delivery systems), surgery (resorbable sutures), and cosmetic applications, as well as for the packaging industry, mainly as disposable products [4,5]. Many research efforts in this area cover various scientific aspects, including the chemical modification of natural polysaccharides, e.g., via substitution (nitration, sulfonation, acylation, alkylation, hydroxylation, phosphorylation, etc.) or grafting copolymerization [3]. The second approach is the synthesis of biodegradable polymers with appropriately designed chemical structures [6]. For example, ring-opening polymerization of cyclic monomers with protective groups allows for us to obtain functional materials capable of furthering post-polymerization modification [7].

To the various biodegradable polymeric materials mentioned above, polymer blends of the appropriate components should be added [8,9].

An example of a biodegradable blend designed for the production of wound dressings, hygiene and cosmetics products, or medical implants is a material based on bacterial cellulose (bionanocellulose) and poly(vinyl alcohol)—BNC/PVA [10,11,12,13]. Research relating to these systems demonstrates that the physicochemical properties required for specific applications can be adjusted by selecting the appropriate production method and composition. The mixture of BNC/PVA combines the good mechanical properties of cellulose with the elasticity of PVA. However, the main advantage of these specimens was their excellent ability to absorb water [12].

In their work on cellulose mixed with poly(vinyl alcohol), other authors pay attention to mechanical properties [14,15,16,17,18]. Moreover, the miscibility of PVA with cellulose and the molecular level’s thermodynamic interactions have also been the subject of research [19,20,21]. The homogeneity of such blends with some microheterogeneity on a nanometer scale has been observed [19].

However, information on the photochemical and thermal stability of such systems is very scarce. Varganici et al. found that poly(vinyl alcohol) underwent slow photooxidative degradation but exhibited a photostabilizing effect on cellulose in PVA–cellulose cryogels containing up to 70% PVA [22]. The main detected processes during UV irradiation of such cryogels were chain scission, photo-oxidation, and decarbonylation, which is in line with generally accepted mechanisms for polymer photodegradation [23,24]. Varganici et al. also studied the thermal stability of PVA–cellulose cryogels [25]. This work determined kinetic parameters and volatile degradation products (mainly H_2_O, CO_2_, olefins, and carbonyl compounds).

Our work aims to study the photochemical and thermal stability of bionanocellulose/poly(vinyl alcohol) blends (BNC/PVA) obtained in three different ways: synthesis in situ, ex situ/impregnation, and ex situ combined with sterilization. For this purpose, the changes in morphology, crystallinity degree, and mechanical and surface properties of UV-irradiated samples were determined; moreover, a thermogravimetric analysis was conducted.

The presented work is a continuation of previous research in which a synthesis methodology and characterization of BNC/PVA composite properties were described in terms of biomedical and cosmetic applications [11,12]. It should be emphasized that these are innovative materials. Due to their application potential, they require testing whether high-energy UV radiation and high temperature can be used as sterilizing agents. According to our knowledge, such mixtures based on biosynthetic cellulose have not yet been presented in the literature.

## 2. Materials and Methods

### 2.1. Materials

Poly(vinyl alcohol), PVA, (30,000–70,000 g/mol and an 87–89% hydrolysis degree) was bought from Aldrich, 3050 Spruce St, St. Louis, MO 63103, USA.

Standard bionanocellulose, BNC, was cultured with bacteria—the *Gluconacetobacter xylinus* E25 strain in Bowil Biotech Ltd., Władysławowo, Poland. Culture medium (Schramm Hestrin) components were obtained from BTL Ltd., Łódź, Poland.

The first 2-day incubation stage, carried out at a temperature of 30 °C and pH of 5.75, was followed by a 7-day stationary synthesis during which a BNC pellicle about 2 mm thick was formed. The obtained BNC material was thoroughly purified with hot water, NaOH solution, 1% CH_3_COOH, and again with water. Thus, obtained BNC served as a reference for the BNC/PVA mixtures, which were prepared by three procedures:

(1) In situ—The culture was carried out according to the method described above for standard BNC, but 1%, 2%, or 4% (*m*/*v*) PVA solution was added to the medium;
(2) Ex situ by impregnation—The originally obtained standard BNC was then soaked in a PVA solution at a concentration of 1%, 2%, and 4% for 2 h, at 80 °C;
(3) Ex situ with sterilization—The samples obtained in the same way as in the second method were additionally sterilized in an autoclave at 121 °C for 20 min.

More details of the preparation and characteristics of the obtained BNC/PVA materials are described in earlier works [11,12,13].

Abbreviations of prepared samples:


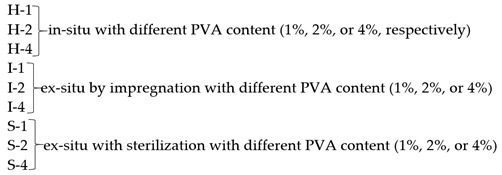


### 2.2. Methods

#### 2.2.1. X-ray Diffraction (XRD)

X-ray diffraction patterns were obtained by the X’Pert PRO (PANalytical, Netherland), using CuKα radiation. The degree of crystallinity (X_c_) of the specimens was estimated by the ratio of the area of the crystalline signals of the bionanocellulose (centered at 2θ 14.0° and 22.1°) to the total area of the XRD curve in the range of 2θ 10–30°.

#### 2.2.2. Atomic Force Microscopy (AFM)

The AFM images of the samples were recorded by using a MultiMode microscope, Veeco Instruments, Inc., Santa Barbara, CA, USA. The scan area was 5 μm × 5 μm and 1 μm × 1 μm. Roughness parameters (the arithmetic mean of the roughness, R_a_; the root mean square average, R_q_; and the maximum distance between the highest and the lowest point on the profile, R_max_ [26]) were calculated from the obtained three-dimensional images.

#### 2.2.3. Contact-Angle Measurement

The contact angle (θ) was measured by a DSA G10 goniometer (Krüss GmbH, Hamburg, Germany), using glycerol and diiodomethane. The result is the average of at least ten measurements for each test liquid. The accuracy of the contact-angle measurement was 2°. The mean θ values were used to calculate the total surface free energy (γ_s_) and its polar (γ_sp_) and dispersion (γ_sd_) components, using the Owens–Wendt method [27].

#### 2.2.4. Mechanical Properties

Mechanical parameters: The breaking stress (σ, MPa), Young’s modulus (E, MPa), and strain at break, i.e., ultimate elongation (ε, %) were determined from a tensile test performed on the modified Instron 1026 machine (Norwood, MA, USA). The wet samples (directly after immersion for 2 h in water) were stretched. The results were obtained for ten replicates. The standard deviation of the breaking stress was 0.4–2.9 MPa, and for the elongation, it was 0.7–3.2%.

#### 2.2.5. Irradiation Conditions

All tested samples were subjected to UV exposure at room conditions, using a bactericidal TUV-30 W lamp (produced by Philips, Holland) emitting 253.7 nm wavelength radiation. The intensity of irradiation at the sample level was 32.2 W/m^2^. The radiation dose that the samples received during 4 h of exposure was 463.68 kJ/m^2^.

#### 2.2.6. Thermogravimetric Analysis

The thermal-stability measurements of the polymer samples were carried out on the Thermal Analysis SDT 2960 Simultaneous DSC-TGA apparatus (USA) in the following conditions: range 20–600 °C, a nitrogen atmosphere, and heating rate of 10 °C/min. From the thermogravimetric curves, the percentage weight loss (Δm); the decomposition onset temperature (T_0_); the temperature at the maximum process speed (T_max_); and the temperatures corresponding to 20%, 50%, and 80% of the weight loss of the tested systems were determined.

## 3. Results

### 3.1. Photoinduced Changes in Sample Crystallinity by XRD

**Figure 1:**
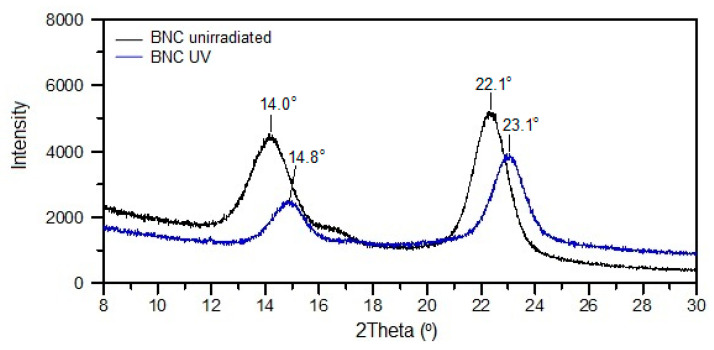
XRD patterns of BNC before and after 4 h of UV irradiation.

The XRD pattern of BNC shows a slight shift of signals toward higher values of 2θ and a drop in intensity resulting from exposure to UV that indicates a decrease in the degree of order in the unmodified biopolymer (Figure 1).

**Figure 2:**
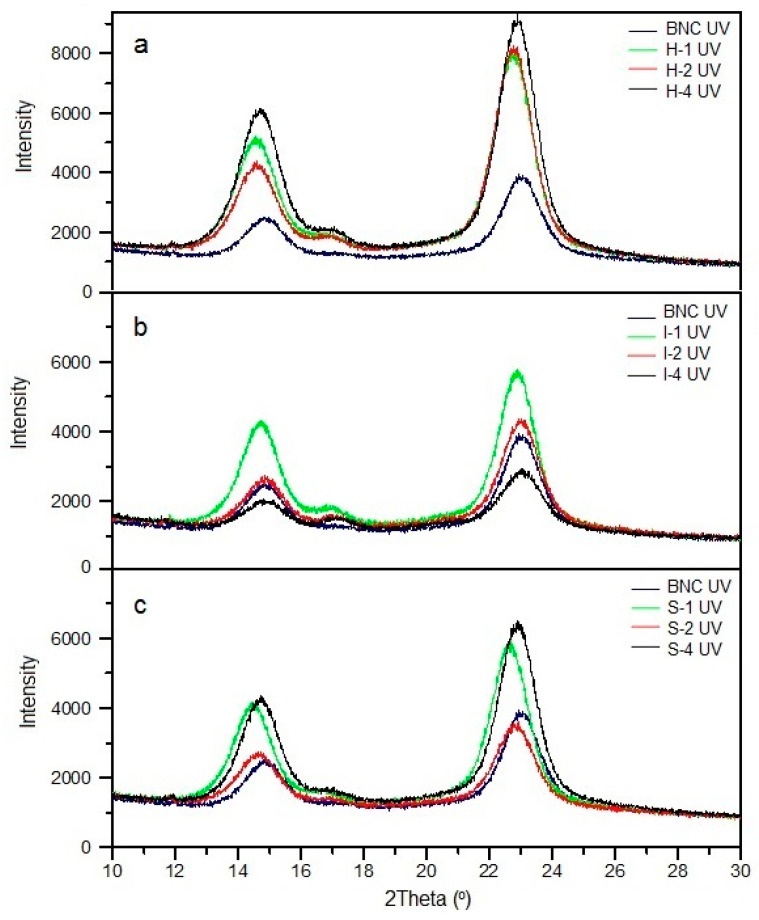
XRD patterns of UV-irradiated BNC/PVA blends obtained by in situ (**a**), ex situ/impregnation (**b**), and ex situ/sterilization (**c**) methods.

The BNC/PVA blends exhibited different changes in X-ray diffraction patterns (Figure 2a–c). BNC (which corresponds to two signals at 2Θ 14.5 and 23° in XRD) is mainly responsible for ordering macromolecules in mixtures, while PVA is the amorphous part. Notably, the broad signal at 2Θ approx. 20°, characteristic for PVA and visible in non-irradiated samples H-1–H-4 and S-4, disappears entirely in XRD after sample exposure to UV. It indicates a preferential photodecomposition of PVA in mixtures.

In UV-treated blends, the intensity of the XRD signals of BNC increases in most cases in the presence of PVA. However, this increase is not proportional to the amount of polymer added to BNC. Only in the materials obtained by in situ cultivation do the signal intensities increase with the concentration of PVA used, and they are the highest among the analyzed systems. We also observe more intense reflections in the XRD of ex situ samples: S-4, S-1, and I-1. The remaining materials show irregular changes.

The full width at half maximum (FWHM) values of two main signals on XRD patterns of BNC and BNC/PVA blends and the differences with these values for non-irradiated samples are listed in Table 1. These results show an increase in FWHM of both main reflections in the XRD caused by UV exposure for most of the analyzed samples (including virgin BNC). The exceptions are the samples I-2 and I-4, for which the FWHM decreases due to the sample irradiation.

The increase in the signal width (FWHM) after sample irradiation indicates that the crystallite sizes of the tested samples are reduced; however, the observed changes are relatively small.

Table 1 also shows the estimated degree of crystallinity (X_c_) of all the samples and the changes from these values for non-irradiated systems (reported in Reference [12]). As can be seen in pure BNC, the X_c_ slightly decreases, while in the blends obtained with all three methods, it increases by several or even a dozen percentage points (1–17%) due to UV radiation. It is due to the photodegradation of the amorphous phase, which is more susceptible to UV radiation than the more compact crystalline part. Thus, it can be concluded that UV radiation causes an etching effect on the surface of BNC/PVA blends.

### 3.2. Changes in Morphology Studied by AFM

AFM microscopy allowed us to assess the surface topography and estimate cellulose fibers’ thickness. The starting BNC and its mixtures with PVA showed a fibrous structure with varying degrees of folding and roughness, in contrast to the relatively smooth PVA surface [12]. After 4 h of UV irradiation, the surface structure observed in the AFM images did not change significantly, which suggests the relative stability of BNC blends. AFM images of UV-irradiated samples are shown in Supplementary Materials Figures S1–S7.

However, the roughness parameters (R_q_, R_a_, and R_max_) changed significantly in UV-irradiated samples (Table 2). The materials obtained in situ (H-1–H-4) due to UV exposure have become rougher compared to non-irradiated systems. The same applies to samples from the ex situ/sterilization method. Additionally, the roughness parameters are the highest in blends, where a 2% PVA solution was used for BNC modification. It is undoubtedly related to the presence of the PVA in the cellulose matrix. In both systems, the poly(vinyl alcohol) is located in the spaces between the BNC fibers; in materials marked H-1–H-4, it is integrally associated with the fibers at the stage of fermentation production. In S-1–S-4 systems, probably a part of the polymer, due to the sterilization process, also penetrates the pores of BNC matrix. Different changes were observed in the composite systems impregnated with PVA—here, a significant part of the polymer was deposited on the outer surface of the BNC. Therefore, in this system, the R_q_, R_a_, and R_max_ parameters decreased after the irradiation process (only I-2 is an exception). This is most likely caused by the surface degradation of the materials, i.e., PVA coating the fibers.

The width of the fibers, as determined from the AFM images, did not change significantly compared to those observed in unexposed systems. Generally, a reduction by a few nanometers in the fibers’ thickness compared to non-irradiated samples was observed; however, the changes are often within the measurement error.

It is difficult to give reliable results of thickness due to the large dispersion of data (from several dozen to over one hundred nm), which is caused by the presence of not-always single fibers. In the AFM images, a large part also consists of bundles of tangled filaments (Supplementary Materials Figures S1–S7).

### 3.3. Changes in Surface Properties Studied by Contact Angle Measurements

The measurement of the contact angle (Θ) is a method sensitive to changes in the surface structure. To evaluate the effect of UV radiation on the obtained materials, the surface free energy and its polar and dispersive components were calculated from determined average Θ values of glycerol and diiodomethane drops deposited on the polymer surface (Table 3).

As previously shown, PVA is characterized by a lower surface polarity despite the hydroxyl groups in the repeating units [12]. It is due to the involvement of the OH groups in hydrogen bonds. As a result of UV radiation, a further decrease in surface free energy and polarity is observed, which is undoubtedly related to the loss of the number of OH groups due to PVA photodegradation. On the contrary, BNC alone exhibits an increase of γ_s_ and its polar component (γ_sp_), which suggests the formation of oxidized functional groups resulting from UV irradiation.

Samples obtained in situ (series H-1–H-4) behave similarly to pure BNC—γ_s_ and γ_sp_ increase after UV exposure. On the other hand, in the blends obtained by the ex situ method, negative changes in γ_sp_ are observed in all cases, as was in the case of PVA itself. It suggests a different structure of these samples—cellulose fibers are surface-coated with PVA.

The lowest surface energy is shown in irradiated samples S-1–S-4; here, also the highest decrease in polarity is observed. It can therefore be concluded that the sterilization of ex situ samples reduces the resistance to UV, mainly PVA.

In all series of samples, the PVA content has no visible influence on the measured parameters. UV radiation causes more remarkable changes in the surface properties of BNC than the addition of PVA.

### 3.4. Changes in Mechanical Properties

**Figure 3:**
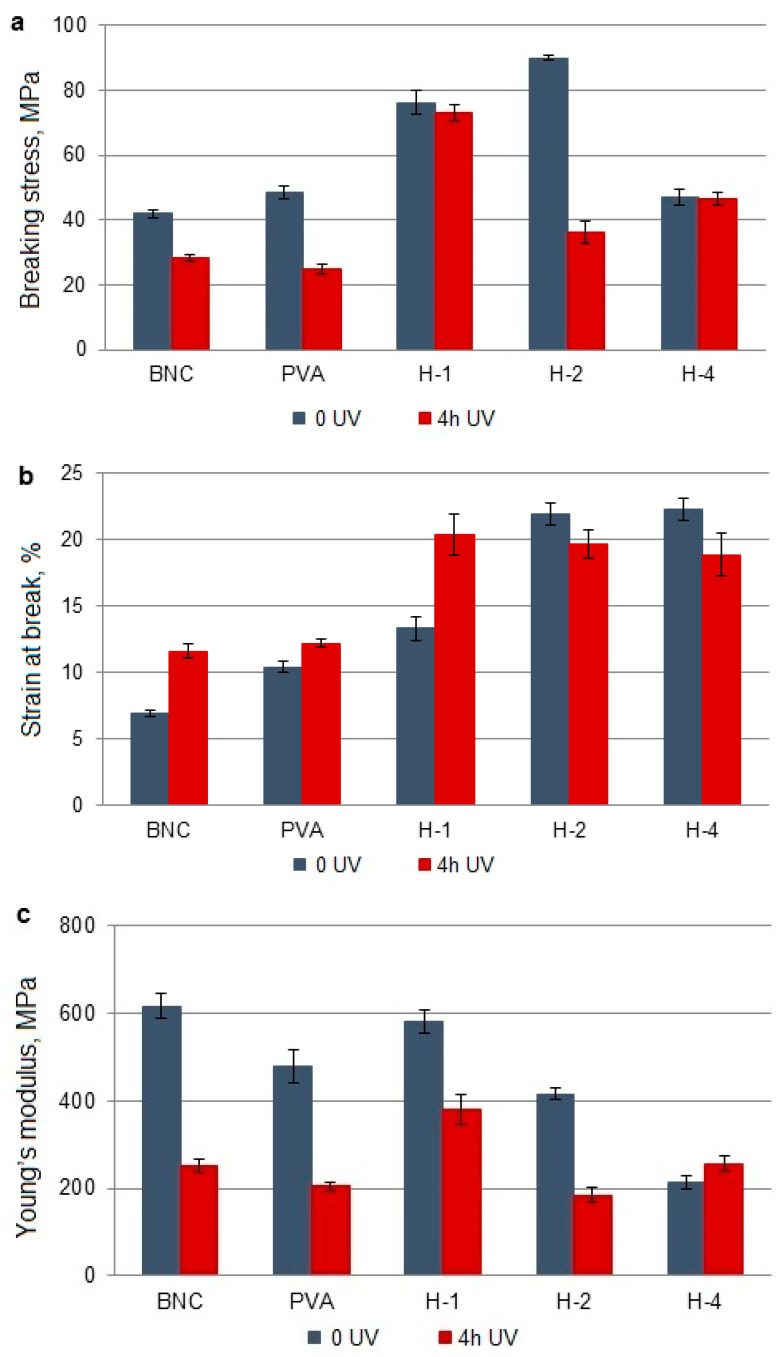
Mechanical properties of BNC, PVA, and their blends obtained by in situ method before and after UV irradiation: breaking stress (**a**), strain at break (**b**), and Young’s modulus (**c**).

**Figure 4:**
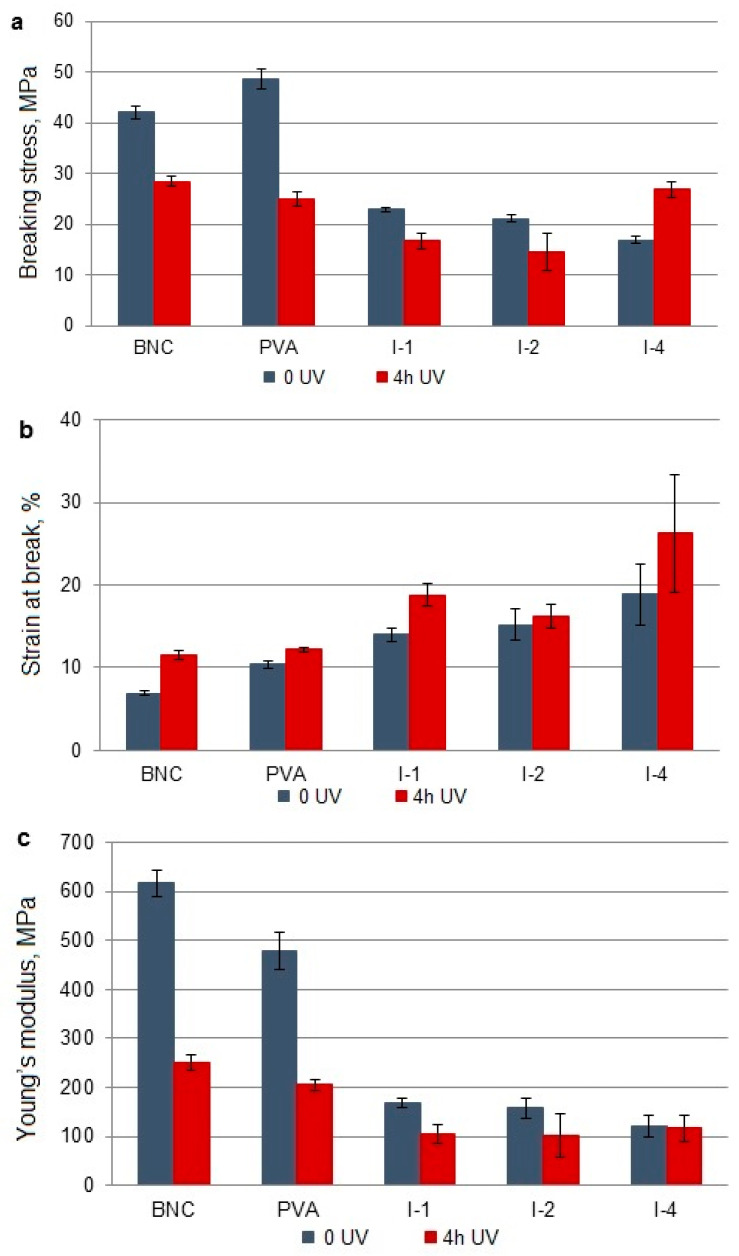
Mechanical properties of BNC, PVA, and their blends obtained by ex situ/impregnation before and after UV irradiation: breaking stress (**a**), strain at break (**b**), and Young’s modulus (**c**).

**Figure 5:**
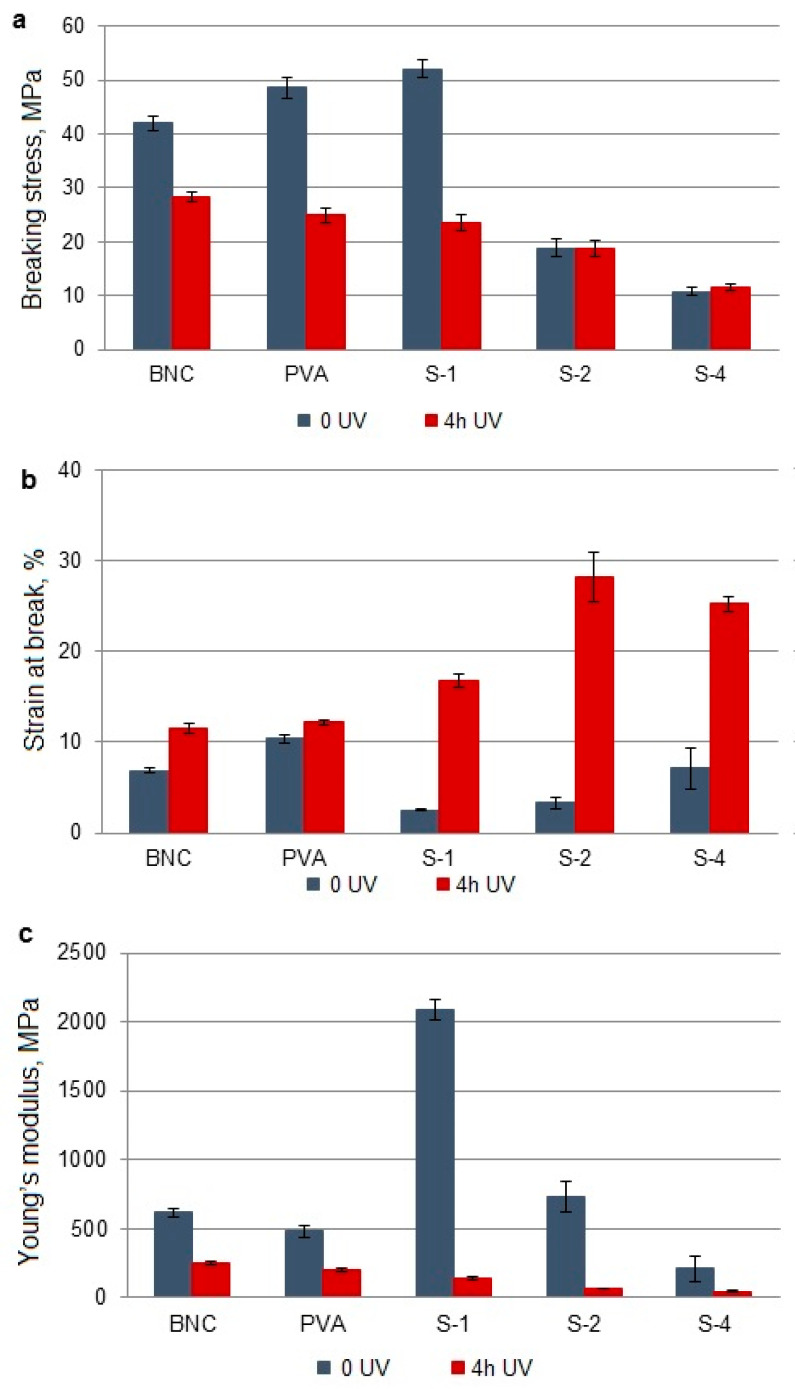
Mechanical properties of BNC, PVA, and their blends obtained by ex situ/sterilization before and after UV irradiation: breaking stress (**a**), strain at break (**b**), and Young’s modulus (**c**).

The influence of UV radiation on the mechanical properties of the obtained BNC/PVA blends and pure polymers is presented in Figure 3, Figure 4 and Figure 5. The breaking stress (σ) and Young’s modulus (elastic modulus, E) in BNC and PVA are nearly half as compared to those values in non-UV-treated materials. Only the strain at break (i.e., relative elongation, ε) increases by 40% in BNC and by 15% in PVA after UV irradiation.

#### 3.4.1. BNC/PVA Obtained In Situ (H-1–H-4)

BNC/PVA blends obtained in situ showed an increase in breaking stress relative to pure BNC and PVA, but this parameter decreases in all samples after 4 h of UV irradiation (Figure 3a). Interestingly, when comparing UV-exposed samples, we see that the σ for H-1–H-4 is higher than that for BNC and PVA alone. The strain at break increases in the mixtures, and this also applies to UV-irradiated samples (Figure 3b). On the other hand, the Young’s modulus is lower in the blend compared to BNC alone, but the opposite trend has been noted after UV treatment (Figure 3c). A relatively high E value was exhibited for H-1.

The deterioration of the mechanical parameters (σ and E) in the irradiated samples proves the main chain scission reactions, while the contrary effect may be caused by competitive crosslinking.

#### 3.4.2. BNC/PVA Obtained Ex Situ/Impregnation (I-1–I-4)

In the case of samples I-1–I-4, the breaking stress values decreased compared to those of pure polymers (Figure 4a). Moreover, due to the UV irradiation of the specimens, σ decreases (the exception is I-4). On the other hand, ε regularly increases with increasing PVA content, and after UV irradiation, it is higher than in unirradiated blends (Figure 4b). The changes in Young’s modulus show a similar trend as σ (Figure 4c).

#### 3.4.3. BNC/PVA Obtained Ex Situ/Sterilization (S-1–S-4)

The system obtained by ex situ/sterilization after UV irradiation shows lower breaking stress values than pure BNC and PVA. Moreover, as the amount of PVA in the system increases, we observe a further decrease in this parameter and Young’s modulus, while the relative elongation increases significantly (Figure 5).

#### 3.4.4. Summary of Changes in Mechanical Properties Due to the Action of UV-Irradiated BNC/PVA Obtained through Ex Situ/Sterilization (S-1–S-4)

To summarize the research results on the mechanical properties of irradiated BNC/PVA samples, the percentage changes of the determined parameters were calculated (Table 4). As the above data show, UV radiation reduces the breaking stress and Young’s modulus of most samples (negative Δσ and ΔE values).

The decrease of Young’s modulus is most significant in samples prepared by ex situ/sterilization (in the range of about 78% to 93%). It proves the higher photodegradation efficiency in these samples. In I-4 and S-4, an increase in σ was observed, suggesting that competing photo-crosslinking processes occur at a higher PVA content in the blend.

The action of UV radiation has a positive effect on the relative elongation of almost all samples. The most significant increase in ε values was found in the systems obtained by ex situ/sterilization (from approx. 253% to 750%). An increase in this parameter resulting from UV exposure can be explained by the plasticizing effect of low-molecular-weight degradation products trapped in the sample matrix.

### 3.5. Thermogravimetric Analysis

A thermal analysis in nitrogen atmosphere was performed on pure polymers, BNC and PVA (as reference samples), and their blends obtained by three methods. The material to be analyzed was thoroughly dried to form a thin polymer film.

**Figure 6:**
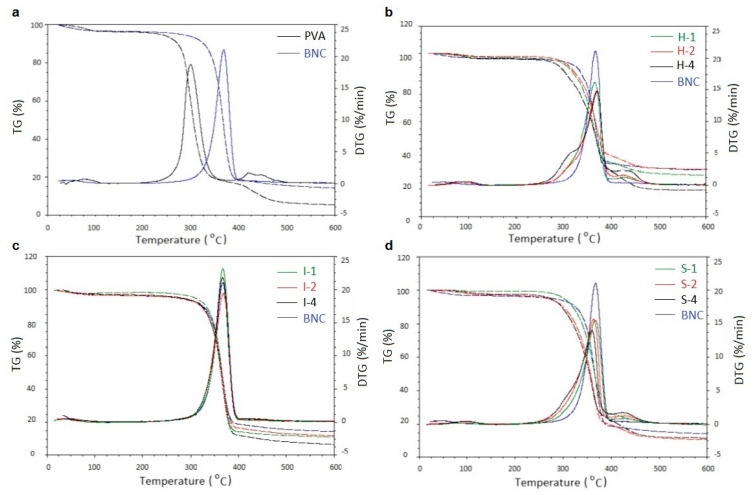
Comparison of TG (dashed lines) and DTG (solid lines) of unmixed BNC and PVA (**a**) materials obtained in situ (**b**), ex situ/impregnation (**c**), and ex situ/sterilization (**d**).

Figure 6a shows the TG and DTG curves of unmixed BNC and PVA, on which we can observe a several-step transformation for both polymers. The first slight loss of cellulose mass in the temperature range of 60–105 °C is related to the evaporation of absorbed moisture (free water and water bound by hydrogen bonds with cellulose fibrils). It constitutes about 4% of the mass of the tested sample.

At a temperature of about 230 °C, we observed the decomposition onset of BNC. In this phase, the supplied thermal energy is sufficient to activate the cleavage of glycosidic bonds and BNC decay (the mechanism described in References [28,29]).

The initial weight loss of PVA in the temperature range of 62–110 °C is also related to the moisture evaporation from this sample (approx. 3%). The second stage, starting at a temperature of 214 °C, corresponds to decomposition into smaller fragments. The final step, which begins at 362 °C, leads to the formation of the carbonized residue. At this stage, as a result of the thermal decomposition of PVA, the following are formed: acetaldehyde, acetone, ethanol, unsaturated aldehydes, ketones, and various aromatic products [30,31]

TG and DTG curves of BNC and its blends with PVA are shown in Figure 6b–d. The characteristic parameters read from these curves are listed in Table 5.

In the case of in situ samples, the thermogravimetric curves show the course of overlapping the processes characteristic for both polymers. Still, the thermal degradation begins at slightly higher temperatures (Figure 6b). Analyzing the mass loss in Stage I, we can see lower Δm values than in BNC itself (except for H-4), and we can see an increase in Δm for Stage II, proportional to the rise in PVA content.

Neglecting the initial water loss, we observed a one-stage thermal decomposition for the system obtained by the ex situ/impregnation method (Figure 6c). Thus, no separate step would be attributed to the degradation of PVA. This phenomenon is probably related to the overlapping of PVA and BNC degradation processes in the same temperature ranges. Confirming the presence of PVA in the BNC matrix is a significant increase in the weight loss of ≥82% (while for cellulose alone, it was 78%). It also means the participation of PVA in the photodegradation process of these blends.

In systems obtained by ex situ/sterilization, we observe the beginning of thermal decomposition at lower temperatures than in BNC. The first stage’s weight loss is much lower than in pure cellulose. It may indicate changes in the course of thermal degradation. The relatively large amount of carbonized residue, which is usually a crosslinked structure, proves the high efficiency of thermal crosslinking in these samples with the participation of poly(vinyl alcohol). Moreover, for these samples (S-1–S-4), the second step of decomposition, characteristic of PVA, appeared on the TG/DTG plots (Figure 6d).

To facilitate the comparison of the thermal stability of the materials obtained with the three methods, the temperatures corresponding to the weight losses of 20%, 50%, and 80% were determined from TG curves (Table 6).

These results indicate that PVA is less thermally stable than BNC. T_20%_ generally decreases with PVA content in all blends, while T_50%_ exhibits very close values to pure BNC. The last parameter, T_80%_, significantly increases in mixtures obtained by in situ and ex situ/impregnation. Thus, considering that T_80%_ corresponds to the second main degradation step, a conclusion can be drawn about a certain mutual stabilization of both polymers in these cases. Ex situ/sterilization samples behave differently, where a slight decrease of the T_80%_ parameter is observed.

## 4. Discussion

### 4.1. General Considerations

There are considerable differences between the photochemical and thermal degradation of polymers (including studied BNC/PVA blends). In the former case, the necessary condition for the reaction to take place is the absorption of the radiation quantum; hence, the photochemical reactions are selective and conditioned by the presence of chromophore groups in molecules [23].

PVA, besides hydroxyl groups, contains carbonyl moieties resulting from incomplete hydrolysis during the synthesis of this polymer from poly(vinyl acetate). They play the role of chromophores.

Bacterial cellulose does not have such radiation-absorbing groups—hence its relatively high resistance to UV. However, even in high-purity cellulose, structural defects can occur. Weak points in macromolecules, even in trace amounts, can contribute to the initiation of photodegradation.

Photodestruction is only possible when the quantum energy is at least equal to the chemical-bond energy. As a result of a homolytic breakage of covalent bonds, free radicals are formed, and it entails successive reactions (so-called dark or secondary) that can take place without radiation [23,32]. Another relevant feature of photochemical processes is their occurrence only in the surface layer of the material. The energy of the radiation quantum with a length of 253.7 nm is 7.835 × 10^−19^ J (4.89 eV), so it is sufficient to break covalent bonds in organic compounds, polymers, and biopolymers.

We should mention that, although FTIR spectroscopy is often used to study the course of photodegradation processes in polymers, in the case of mixtures consisting of components having the same functional groups (OH), their differentiation in infrared spectra is impossible. Furthermore, detecting the photo-oxidized groups accompanying their detachment during photodegradation is difficult. An additional difficulty is the lack of transparency of the BNC/PVA systems, which means that only reflection techniques can be used, which are not suitable for quantitative research. Therefore, we present a different approach here by examining the influence of UV radiation on the morphology and functional blend properties.

Thermal degradation is not limited to the material surface, as heat is applied simultaneously to all molecules in the whole sample volume. In the initial stages of heating, the molecules gain more and more kinetic energy, which can lead to phase changes. Finally, at a sufficiently high temperature, they decompose. The degradation process begins with breaking the weakest bonds, followed by an intensive release of degradation products of different volatility and chemical composition [33,34].

Both processes (photochemical and thermal) depend on the environment in which they occur. In our experiment, the UV irradiation took place in the air atmosphere; hence, oxidation reactions accompanied the degradation processes. Thus, we are dealing with photo-oxidative degradation. On the other hand, the thermogravimetric analysis was carried out in dynamic conditions but in an inert atmosphere (N_2_); hence, oxidation is excluded here. The decomposition process observed in such conditions provides information not only about thermal stability but also about the composition and chemical structure of the tested systems.

### 4.2. Brief Mechanism of Thermal and Photochemical Degradation of BNC/PVA Blends

The mechanism of the processes taking place in heated or UV-irradiated BNC/PVA blends can be explained on the base of processes occurring in degraded BNC and PVA separately [23,35]. The initiation of macromolecule decay is associated with forming free radicals, which are highly active and cause complex secondary reactions.

The first step in the thermal degradation of cellulose is the removal of physically bound water. Then glycoside linkages (C_1_∓C_4_) break, OH and CH_2_OH groups are abstracted, and saccharide rings open. The cellulose chains are transformed into relatively low-molecular-weight oligosaccharides and even monosaccharides. An example of an anhydrous monosaccharide obtained during the thermal decomposition of cellulose is levoglucosan, which can undergo further dehydration and isomerization, forming various derivatives [28,29] during decarbonylation and decarboxylation, as well as in more complex processes, with the release of other volatile organic products.

Thermal degradation of PVA starts with the elimination of water due to the abstraction of the side groups, resulting in the formation of polyene structures. Residual acetate groups are also eliminated, hence the presence of relatively large amounts of acetic acid during degradation [30]. The decomposition temperature in the first degradation step is not high enough to generate other low molecular weight products. Initially, intermediates are formed, which further decompose at higher temperatures.

The second stage of decomposition is the continuation of the detachment of side groups and the intensive breakage of the main chains, accompanied by reactions of degraded molecules, e.g., intramolecular cyclization or crosslinking. The degradation products mainly include acetaldehyde, acetic acid, polyethers, aromatic derivatives, and a small amount of furan [31,32].

In UV-irradiated BNC/PVA blends, carbonyl group excitation leads to Norrish reactions and secondary processes [23], mainly chain scission and abstraction of side substituents. The network of inter- and intra-molecular hydrogen bonds is also destroyed. Any radical or macroradical (P^∙^) resulting from the homolytic breaking of the covalent bond can react with atmospheric oxygen to form an unstable peroxide radical (P-O-O^∙^). Then, as a result of attaching a hydrogen atom from the neighboring unit, these radicals transform into hydroperoxide groups (P-OOH), which further decompose into alkoxyl (P-O^∙^) and hydroxyl radicals (HO^∙^). These reactions are chain processes and lead to the formation of various oxidized products (i.e., ketone, aldehyde, and carboxyl groups) and unsaturated chain ends [32,35].

The recombination of the radicals deactivates them and terminates the chain of degradation. Moreover, the recombination of macroradicals is the reason for crosslinking. One should remember the possibility of interaction and chemical reactions of free radicals and degradation products formed in one polymer with macromolecules of the other component, and vice versa [36]. Indeed, many competing reactions coincide during the thermal and photochemical degradation of polymeric blends, and their result impacts the final properties of materials.

We found that UV-irradiated BNC/PVA materials did not degrade very efficiently, as only a slight influence on the morphology, surface, and mechanical properties, as well as the order of the macromolecules, was observed.

## 5. Conclusions

In this work, novel BNC/PVA composites with different PVA shares, obtained in the biosynthetic process (i.e., in situ) or based on bacterial cellulose in the impregnation or additional sterilization (ex situ) procedures, were characterized in terms of their photochemical and thermal resistance.

After UV irradiation, the degree of crystallinity in all BNC/PVA blends slightly increases, and this is caused by the photodegradation of the amorphous phase (mainly PVA). The surface roughness also increases (except I-1 and I-4) as a result of of photo-etching. The polarity depends on the preparation method—it grows in the irradiated samples in situ, while it decreases in both types ex situ. Moreover, the mechanical properties depend on the method used. A decrease in the strength parameters (σ, E) was observed due to photodegradation, while the relative elongation increased, except for samples H-2 and H-4. It can be due to random chain scission and the release of shorter chain fragments acting as a plasticizer.

The thermal stability of BNC/PVA blends results from competitive reactions: degradation and crosslinking. A minor increase in thermal stability was mainly observed in the samples obtained in situ.

In summary, the obtained materials meet the requirements for practical applications in medicine, pharmacy, and cosmetology. These studies showed that the functional properties do not deteriorate significantly under UV radiation, and the thermal stability is also satisfactory. Considering the major thermal-degradation step, both polymers were even found to be mutually stabilized. Thus, these two factors—short-wavelength UV radiation and high temperature (up to at least 200 °C)—can be alternative sterilizing agents. Such behavior of BNC/PVA blends has not yet been described in the contemporary scientific literature.

Moreover, taking into account the previously described properties [12]—high water absorption and good mechanical resistance—the composites obtained by the in situ method are good candidates for implantation, while the systems from the ex situ/sterilization method are suitable for production a wound dressing with little exudation. On the other hand, the mixture produced by the ex situ/impregnation may be primary material for cosmetic applications or a dressing for wounds with high exudate. In addition, the good thermal resistance of all tested samples allows for other applications in high-temperature conditions, including modern technologies in the automotive, aviation, marine, microelectronic, and chemical industries.

## Figures and Tables

**Table 1:** The full width at half maximum (FWHM, °) of XRD signals and degree of crystallinity (X_c_, %) of UV-irradiated BNC and BNC/PVA blends. The difference between the values in relation to the non-irradiated samples is given in brackets ^1^.

Sample	2θ ~ 14.5°	2θ ~ 23°	X_c_, %
BNC	1.42 (+0.10)	1.43 (+0.11)	39 (−2)
H-1	1.42 (+0.12)	1.45 (+0.20)	44 (+7)
H-2	1.42 (+0.18)	1.41 (+0.10)	44 (+9)
H-4	1.40 (+0.22)	1.43 (+0.26)	45 (+17)
I-1	1,32(+0.09)	1.39 (+0.09)	44 (+7)
I-2	1.30 (−0.05)	1.41 (+0.14)	42 (+6)
I-4	1.32 (−0.18)	1.45 (+0.14)	37 (+1)
S-1	1.38 (+0.01)	1.38 (+0.4)	41 (+4)
S-2	1.40 (+0.14)	1.44 (+0.16)	40 (+4)
S-4	1.39 (+0.14)	1.42 (+0.12)	44 (+9)

^1^ The corresponding values for unirradiated samples are presented in Reference [12].

**Table 2:** Parameters R_q_, R_a_, and R_max_ of UV-treated BNC and BNC/PVA blends obtained by various methods. The difference between the values in relation to the non-irradiated samples is given in parentheses ^1^.

Sample	R_q_, nm	R_a_, nm	R_max_, nm
BNC	46.6 (+15.9)	38.3 (+14.0)	297 (+188)
H-1	37.0 (+14.2)	29.3 (+11.1)	322 (+208)
H-2	67.6 (+41.3)	53.3 (+32.4)	426 (+146)
H-4	46.2 (+28.8)	36.8 (+23.2)	333 (+197)
I-1	46.8 (−25.3)	37.9 (−17.4)	336 (−306)
I-2	96.3 (+53.3)	77.1 (+43.6)	649 (+489)
I-4	39.9 (−19.7)	31.7 (−15.0)	276 (−69)
S-1	39.2 (+9.40)	31.3 (+7.70)	272 (+112)
S-2	82.9 (+46.3)	64.9 (+36.2)	646 (+504)
S-4	47.3 (+12.0)	37.0 (+8.80)	485 (+293)

^1^ The corresponding values for unirradiated samples are presented in Reference [12].

**Table 3:** Total surface free energy (γ_s_) and its dispersion (γ_sd_) and polar (γ_sp_) components of 4 h UV-irradiated BNC and BNC/PVA blends obtained by various methods. The differences between the values with the non-irradiated samples are given in parentheses ^1^.

Sample	γ_s_, mJ/m^2^	γ_sd_, mJ/m^2^	γ_sp_, mJ/m^2^
BNC	46.22 (+2.31)	28.58 (−0.32)	17.64 (+2.63)
PVA	23.07 (−10.06)	22.18 (−3.26)	0.89 (−6.80)
H-1	43.28 (+0.45)	29.99 (+0.17)	13.29 (+0.27)
H-2	48.29 (+4.14)	25.84 (−3.73)	22.45 (+7.87)
H-4	50.78 (+3. 21)	31.35 (0.00)	19.43 (+3.21)
I-1	47.89 (+0.34)	34.45 (+3.65)	13.44 (−3.31)
I-2	41.80 (−6.97)	31.84 (−1.19)	9.96 (−5.78)
I-4	40.02 (−9.03)	29.86 (−3.05)	10.16 (−5.98)
S-1	38.26 (+2.90)	33.03 (+11.93)	5.23 (−9.03)
S-2	40.40 (+2.70)	36.88 (+14.45)	3.52 (−11.75)
S-4	31.95 (−7.44)	27.81 (+4.00)	4.14 (−11.45)

^1^ The corresponding values for unirradiated samples are presented in Reference [12].

**Table 4:** Changes in strength parameters (%) caused in BNC and PVA samples and BNC/PVA blends after 4 h UV irradiation.

Sample	Δσ, %	Δε, %	ΔE, %
BNC	−32.41	+68.13	−59.45
PVA	−48.61	+17.21	−57.29
H-1	−3.85	+52.66	−34.47
H-2	−59.89	−10.37	−55.58
H-4	−1.09	−15.35	+19.56
I-1	−26,88	+33,54	−37,25
I-2	−30,54	+6,44	−36,18
I-4	+59,42	+39,30	−3,71
S-1	−54.73	+570.35	−93.27
S-2	−0.62	+749.90	−90.69
S-4	+7.51	+252.65	−77.93

**Table 5:** Thermal parameters determined from the TG and DTG curves of all tested samples.

Sample	T_o_, °C	T_max_, °C/Δm, %	
		I Step	II Step
BNC	230	367/78	-
PVA	214	298/78	427/12
H-1	235	367/75.8	430/9.0
H-2	234	370/71.0	431/12.0
H-4	233	372/78.4	427/18.6
I-1	219	367/86.0	-
I-2	209	368/82.0	-
I-4	204	366/88.0	-
S-1	229	368/72.0	427/7.0
S-2	211	363/74.4	426/11.0
S-4	214	361/70.4	425/14.8

**Table 6:** The temperatures corresponding to the weight losses of 20%, 50%, and 80% determined from TG curves.

Sample	T_20%_, °C	T_50%_, °C	T_80%_, °C
BNC	347	366	388
PVA	287	304	343
H-1	332	361	397
H-2	337	368	432
H-4	317	359	382
I-1	340	366	555
I-2	326	357	395
I-4	320	355	415
S-1	348	366	381
S-2	343	365	384
S-4	343	364	379

## Data Availability

The data presented in this study are available on request from the corresponding author.

## Supplementary Materials

The following supporting information can be downloaded at https://www.mdpi.com/article/10.3390/polym14204364/s1. Figure S1: AFM images of 4 h UV-irradiated BNC; Figure S2: AFM imagesI of 4 h UV-irradiated H-2 sample; Figure S3: AFM images of 4 h UV-irradiated H-4; Figure S4: AFM images of 4 h UV-irradiated I-2; Figure S5: AFM images of 4 h UV-irradiated I-4; Figure S6: AFM images of 4 h UV-irradiated S-2; Figure S7: AFM images of 4 h UV-irradiated S-4.
