# Supplementary material for: Photochemical and Thermal Stability of Bionanocellulose/Poly(Vinyl Alcohol) Blends
Source: Polymers (Basel). 2022 Oct 16;14(20):4364. doi: 10.3390/polym14204364 (PMC9609272; doi:10.3390/polym14204364)
Supplement: Supplementary file 1 [file polymers-14-04364-s001.zip › polymers-1938777-Supplementary.pdf]

Supplementary Materials

Article

Photochemical and Thermal Stability of Bionanocellulose/Poly(Vinyl Alcohol) Blends

Aldona Długa [1,†], Dagmara Bajer [2,*] and Halina Kaczmarek [2,*]

[1] Bowil Biotech Sp. z.o.o., 7 Skandynawska St., 84-120 Władysławowo, Poland
[2] Faculty of Chemistry, Nicolaus Copernicus University in Toruń, 7 Gagarina St., 87-100 Toruń, Poland
* Correspondence: dagmara@umk.pl (D.B.); halina@umk.pl (H.K.); Tel.: +48-56-6114-502 (D.B.); +48-56-6114-312 (H.K.)
† Current address: Blirt S.A., 3 Trzy Lipy St., 80-172 Gdańsk, Poland.

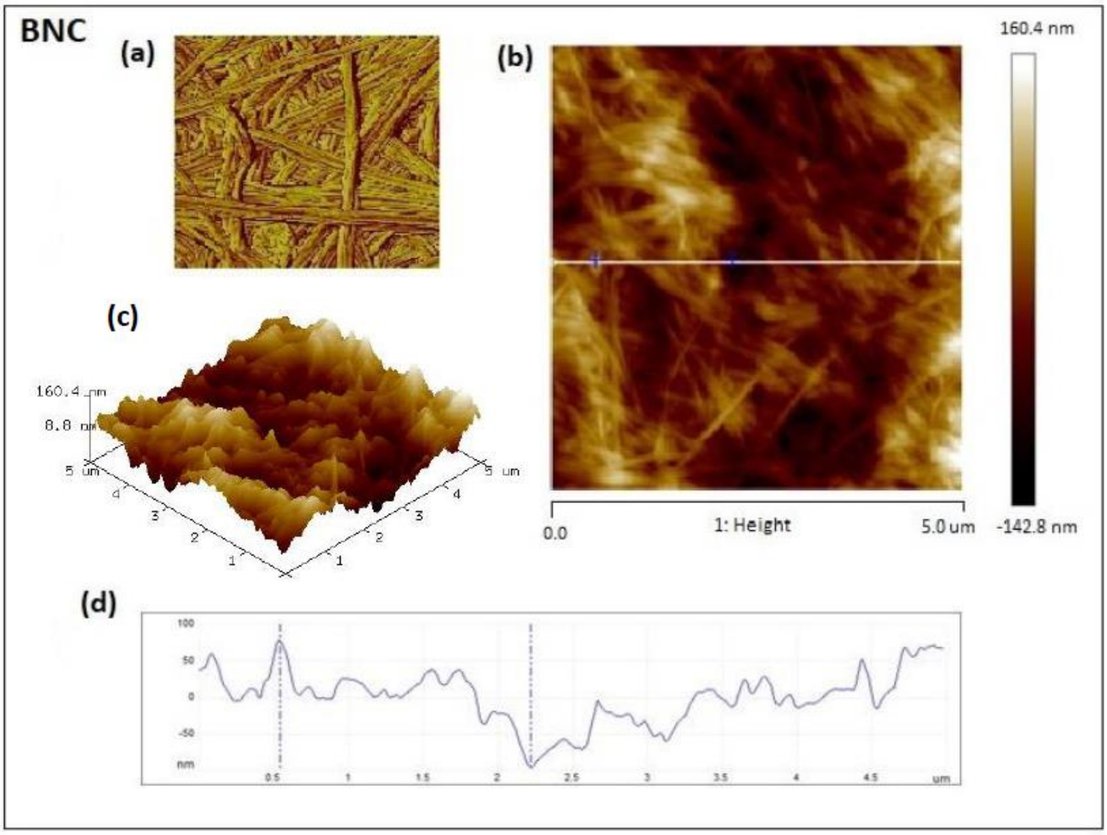

Figure S1. AFM images of 4h UV-irradiated **BNC**: phase - 1μm x 1μm (a), height (b), semi-3D (c), and cross-section of the surface along the line indicated in Fig. b (d).

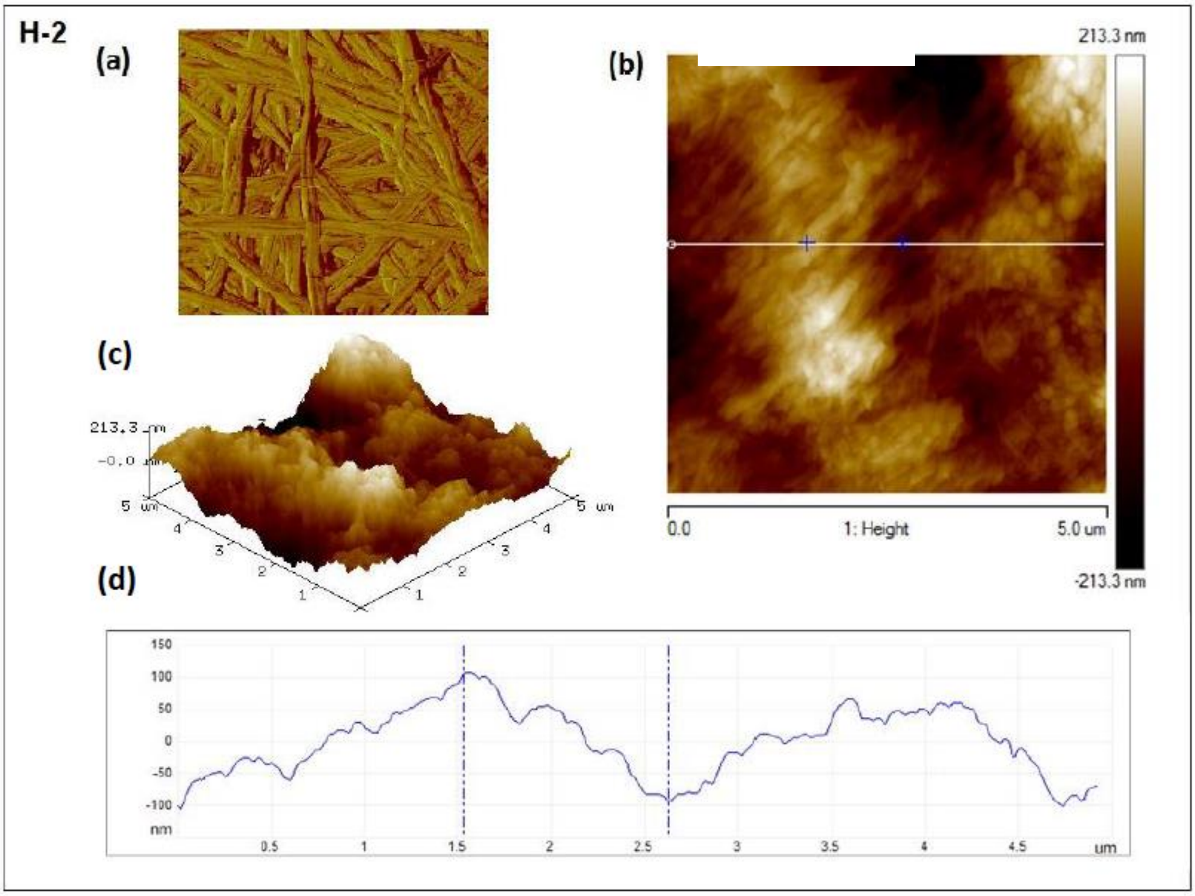

Figure S2. AFM images of 4h UV-irradiated **H-2** sample: phase - 1μm x 1μm (a), height (b), semi-3D (c), and cross-section of the surface along the line indicated in Fig. b (d).

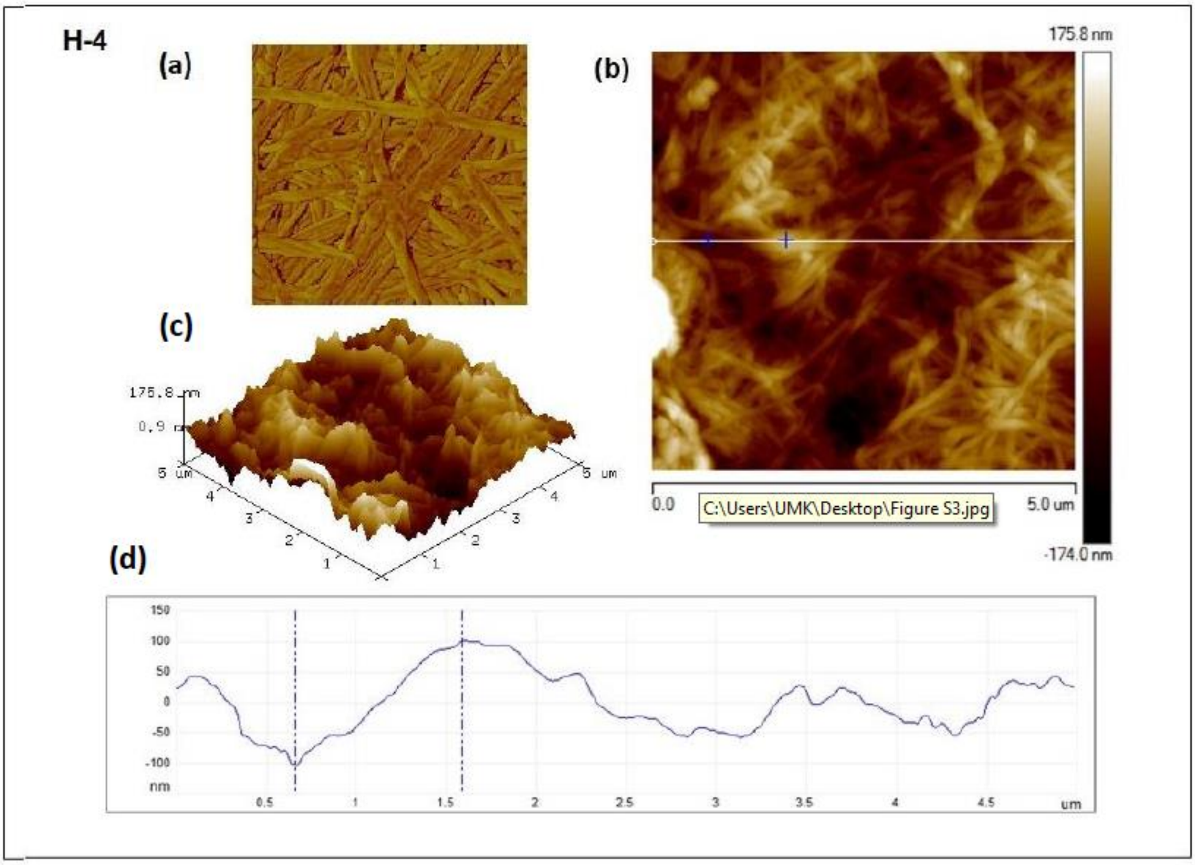

Figure S3. AFM images of 4h UV-irradiated **H-4**: phase - 1μm x 1μm (a), height (b), semi-3D (c), and cross-section of the surface along the line indicated in Fig. b (d).

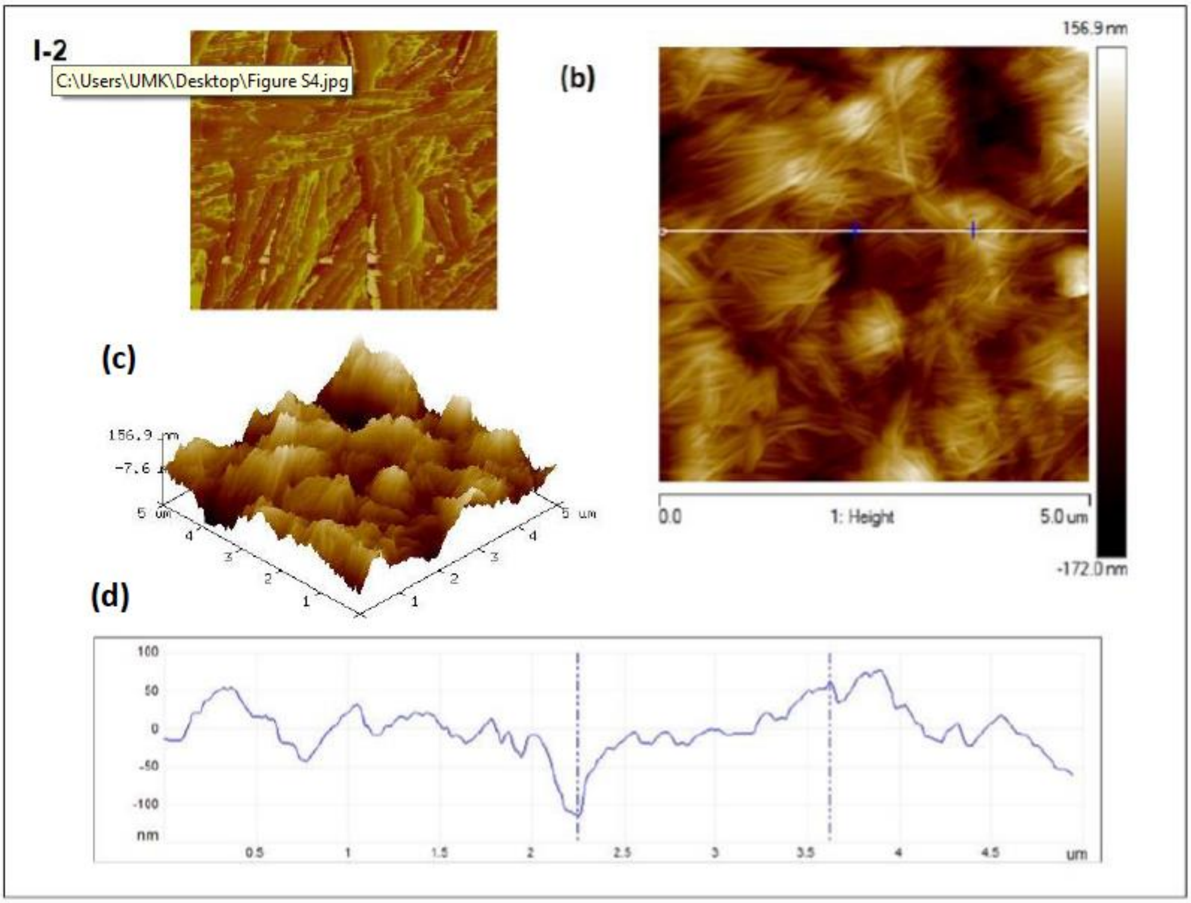

Figure S4. AFM images of 4h UV-irradiated **I-2:** phase - 1μm x 1μm (a), height (b), semi-3D (c), and cross-section of the surface along the line indicated in Fig. b (d).

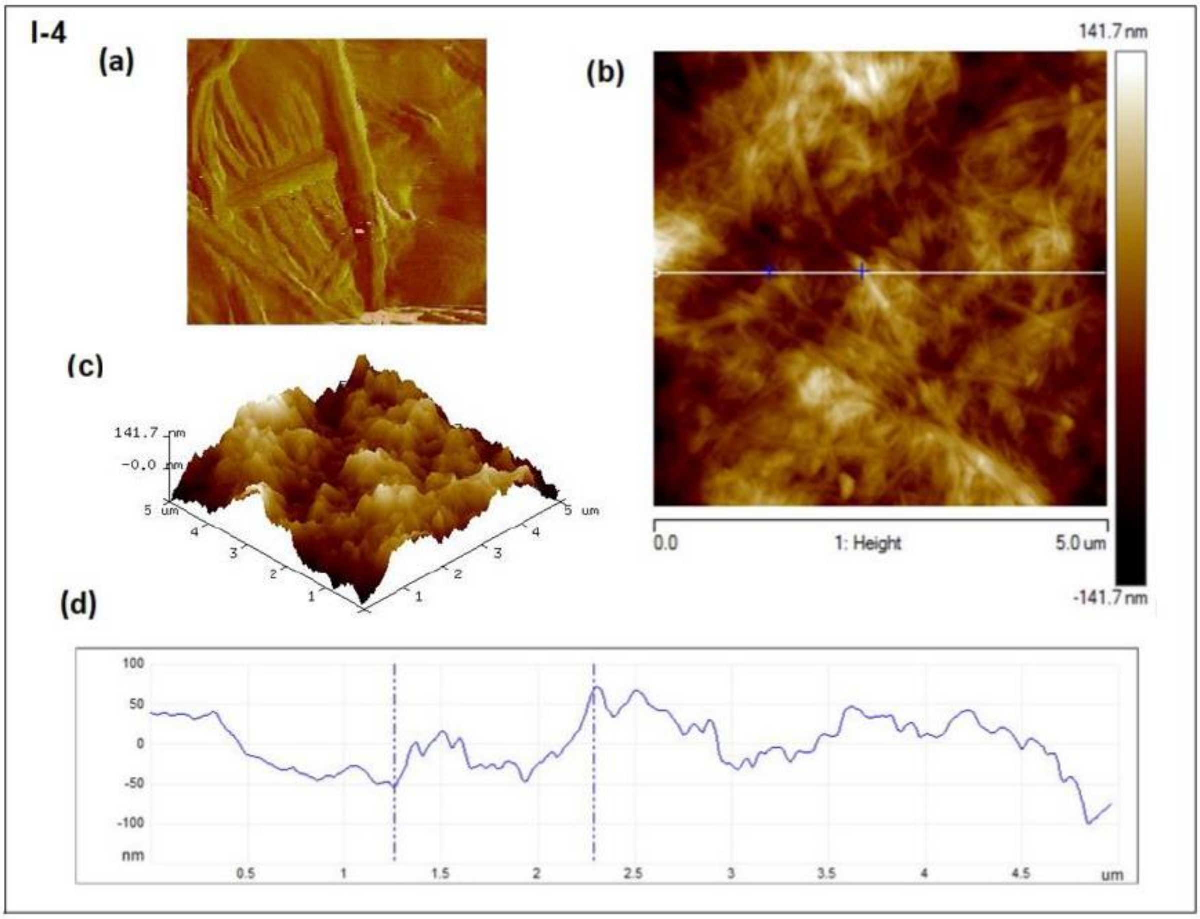

Figure S5. AFM images of 4h UV-irradiated **I-4**: phase - 1μm x 1μm (a), height (b), semi-3D (c), and cross-section of the surface along the line indicated in Fig. b (d).

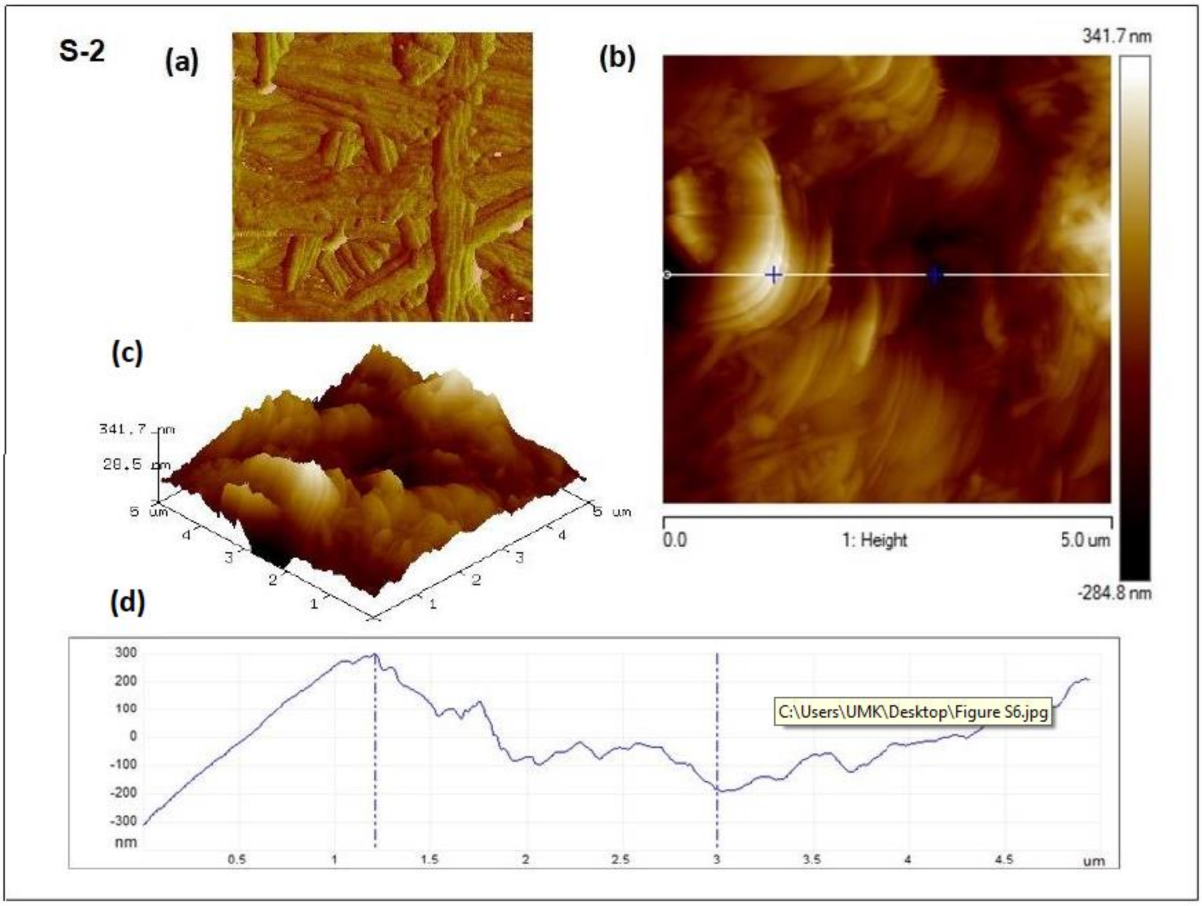

Figure S6. AFM images of 4h UV-irradiated **S-2**: phase - 1μm x 1μm (a), height (b), semi-3D (c), and cross-section of the surface along the line indicated in Fig. b (d).

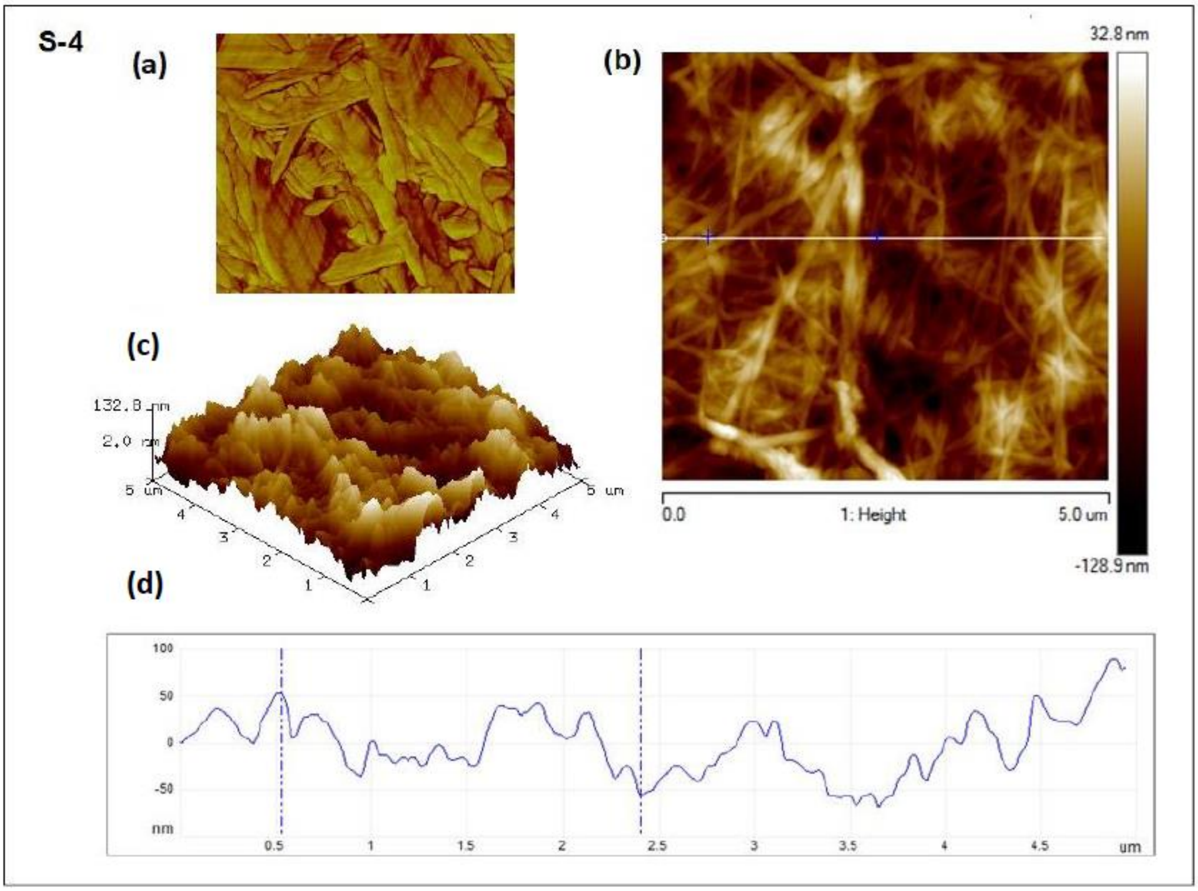

Figure S7. AFM images of 4h UV-irradiated **S-4**: phase - 1μm x 1μm (a), height (b), semi-3D (c), and cross-section of the surface along the line indicated in Fig. b (d).
